# The Phenolic Components of *Gastrodia elata* improve Prognosis in Rats after Cerebral Ischemia/Reperfusion by Enhancing the Endogenous Antioxidant Mechanisms

**DOI:** 10.1155/2018/7642158

**Published:** 2018-03-22

**Authors:** Anhuan Shi, Jianming Xiang, Fangyan He, Yanping Zhu, Gongbei Zhu, Yuhan Lin, Ningna Zhou

**Affiliations:** ^1^Department of Pharmacology, Yunnan University of Traditional Chinese Medicine, Kunming, China; ^2^Department of Neurosurgery, Medical School, University of Michigan, Ann Arbor, MI, USA

## Abstract

Pharmacological or spontaneous thrombolysis in ischemic stroke triggers an outbreak of reactive oxygen species and results in neuron death. Nrf2-mediated antioxidation in cells has been proved as a pivotal target for neuroprotection. This research reports that phenolic components of *Gastrodia elata* Blume (PCGE), a traditional Chinese medicine, can alleviate the pathological lesions in the penumbra and hippocampus by increasing the survival of neurons and astrocytes and improve neurofunction and cognition after reperfusion in a rat model of middle cerebral artery occlusion. LDH assay indicated that pretreatment of cells with PCGE (25 *μ*g/ml) for 24 h significantly reduced H_2_O_2_-induced cell death in astrocytes and SH-SY5Y cells. Western blot showed that the nucleus accumulation of Nrf2 and the expression of cellular HO-1 and NQO-1, two of Nrf2 downstream proteins, were increased in both cells. BDNF, an Nrf2-dependent neurotrophic factor, was also upregulated by PCGE in astrocytes. These results illustrated that PCGE can reduce the cerebral ischemia/reperfusion injury and improve prognosis by remedying the cell damage within affected tissues. The protective effects of PCGE seem to be via activation of a Nrf2-mediated cellular defense system. Therefore, PCGE could be a therapeutic candidate for ischemic stroke and other oxidative stress associated neurological disorders.

## 1. Introduction

Embolic or atherothrombotic blocking to a cerebral artery is defined as ischemic stroke and accounts for 80% of all stroke cases. Tissue plasminogen activator (tPA) approved by the US Food and Drug Administration (FDA) in 1996 is still the only pharmacological intervention for acute ischemic stroke. However, due to narrow eligibility and treatment windows, only 2%–5% of all stroke patients receive tPA treatment and only 50% of that achieve successful reperfusion [1]. Furthermore, tPA intervention increases the risk of intracerebral hemorrhage [2] and reperfusion injury [3]; thus, the application of tPA in the clinic has been limited. Neuronal apoptosis or necrosis is an inevitable result of ischemic injury, which subsequently induces limb, language, and cognitive dysfunction, and remains a tough challenge for therapeutics. A recent analysis of census data hints that the incidence of ischemic stroke will be doubled over the next 40 years [4]. Therefore, the exploration of potential therapeutic agents that may lead to a viable clinical application is needed urgently [5].

Extensive research has been carried out on the contribution of oxidative stress to neuron death in stroke [6–8]. On account of a high speed of oxidative metabolic activity and insufficient neural restoration competence, the brain is very impressionable to damage induced by oxidation during ischemia [9]. The allegro accumulation of reactive species including oxygen (ROS) and nitrogen (RNS) after acute ischemic stroke exceeds endogenous antioxidant defense mechanisms and results in energy impairment, mitochondrial dysfunction, accumulation of aggregated proteins, and alterations in homeostasis. These so-called ischemic cascade events contribute to neuron death by apoptotic, necrotic, and autophagic mechanisms. Additionally, a second breakout of ROS generation after the rapid restoration of blood flow caused by pharmacological or spontaneous thrombolysis accounts for the reperfusion injury [10, 11]. Therefore, antioxidative stress has been investigated as a strategy to attenuate the injury of ischemic stroke. It has been reported that antioxidants can reduce tissue levels of the superoxide anion and limit progression of brain infarction [12].

*Gastrodia elata* Blume, a traditional Chinese medicine, has been proved to have therapeutic effects on cardio- and cerebral-vascular diseases, and several of its formulae have been applied to acute cerebral infarction and hypertension in the clinic [13–15]. Although previous study of our laboratory has shown that the phenolic components of *G*. *elata* (PCGE) can protect the brain from cerebral ischemia via inhibition of nitric oxide synthase (NOS) pathways [16], the related mechanism is still unknown. It is of importance to explore the effect and mechanism of PCGE as a novel therapeutic agent for neurological disorders. We therefore hypothesized that PCGE can remedy the neurons from cerebral ischemia/reperfusion by activating the endogenous antioxidative system. The neuroprotective effect of PCGE was determined by a transient middle cerebral artery occlusion model in rat, and its molecular mechanisms were determined in the human primary astrocyte HA-1800 and SH-SY5Y cells, a human neuroblastoma cell line.

## 2. Materials and Methods

### 2.1. Materials

Fetal bovine serum (FBS) was purchased from Hyclone (Thermo Scientific, China). Dulbecco's modified Eagle's medium (DMEM), L-glutamine, Antibiotic-Antimycotic (100X), trypsin-EDTA, H_2_O_2_, and collagen were obtained from Invitrogen (Life Technologies, China).

PCGE preparation: the rhizomes of *Gastrodia elata* Blume were purchased from Zhaotong, Yunnan province, China. PCGE was prepared as described previously by our lab [17]. Briefly, a dried and ground rhizome of *G*. *elata* was treated with 70% ethanol by maceration, and the ethanol extraction was concentrated under decompression. The concentrate was then resuspended in distilled water and further extracted repeatedly with ethyl acetate. The fraction generated from the ethyl acetate extraction was used as PCGE. The extraction ration of PCGE is around 1.14%. In this study, PCGE were dissolved in either 1% Tween-80 for *in vivo* study or dimethylsulfoxide (DMSO) for *in vitro* study.

The fingerprint analysis of the PCGE sample was performed by high-performance liquid chromatography (HPLC) as described with slight modification [18]. Four standard substances (gastrodin, 4-hydroxybenzaldehyde, 4-hydroxybenzylalcohol, and 3,4-dihydroxybenzaldehyde) were purchased from the J&K Scientific Ltd. (Beijing, China). The tested sample was dissolved in 3% acetonitrile, and the solution was filtered using a 0.45 *μ*m Millipore® filtration unit. The HPLC system (Agilent 1260 Infinity II, Agilent Technologies, China) was equipped with a binary solvent delivery system and an autosampler. 10 *μ*l of the sample was separated on a Zorbax SB-C18 column (4.6 mm × 250 mm, 5 *μ*m; Agilent Technologies, China) maintained at 25°C. The mobile phase consisted of acetonitrile and 0.1% aqueous acetic acid in isocratic elution mode with the ratio of 3 to 97 (*v*/*v*). The flow rate was fixed at 1.0 ml/min, and the DAD detector was set at 270 nm.

**Figure 1:**
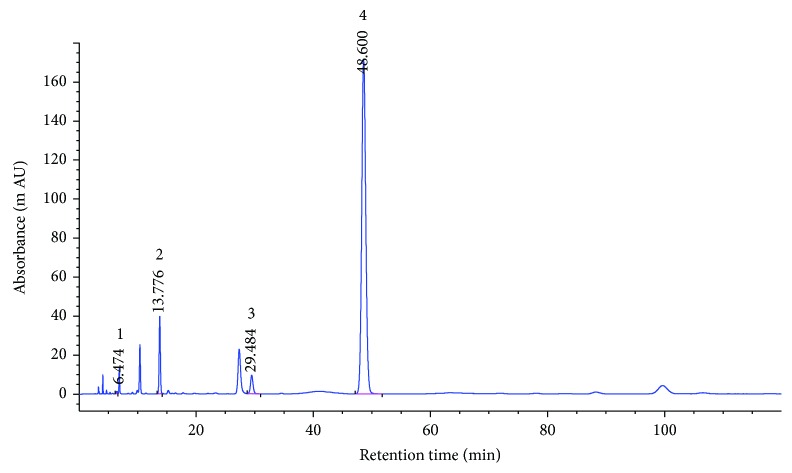
HPLC-fingerprint analysis of PCGE. 1, gastrodin; 2, 4-hydroxybenzylalcohol; 3, 3,4-dihydroxybenzaldehyde and 4, 4-hydroxybenzaldehyde.

The PCGE-fingerprints (Figure 1) were characterized by two dominating peaks of 4-hydroxybenzaldehyde (peak 4, Rt = 48.60) and 4-hydroxybenzylalcohol (peak 2, Rt = 13.78), with much lower contents of gastrodin (peak 1, Rt = 6.47) and 3,4-dihydroxybenzaldehyde (peak 3, Rt = 29.48) according to the standard substance fingerprint. The content of these 4 compounds in PCGE was calculated as the peak area of the compound relative to the total peak area (Table 1).

### 2.2. Cerebral Ischemia/Reperfusion in Rats

The protocols for these animal studies were approved by the Committee of Yunnan University of Traditional Chinese Medicine in the Use and Care of Animals (permit number: R-06001). Adult male Sprague-Dawley rats weighing between approximately 280 g and 300 g were fasted overnight before surgery with free access to water. General anesthesia was induced with an intraperitoneal injection of chloral hydrate (300 mg/kg, Chengdu Kelong Chemical Reagent Factory, China). Body temperature was maintained at 37°C with a feedback-controlled heating pad (JR-1/2, Chengdu TME Technology Co. Ltd., China) during the whole procedure.

Ischemia were induced by transient middle cerebral artery occlusion (MCAO) which was performed as reported previously [19] with slight modifications. Briefly, the left common carotid artery (CCA), internal carotid artery (ICA), and external carotid artery (ECA) were surgically exposed, and then the branches of the ECA (occipital artery) and the ICA (pterygopalatine artery) were coagulated. The ECA and the CCA were ligated with a 3-0 surgical silk suture, and the ICA was distally clamped with a microvascular clip. A 3-0 nylon suture with rounded tip was inserted into the ICA through the ECA stump and gently advanced until mild resistance was encountered (about 18–20 mm from the bifurcation of the ECA and the ICA to the initiation of the middle cerebral artery) to occlude the middle cerebral artery (MCA). After 2 h of focal ischemia, the suture was withdrawn and the common carotid artery was reopened for reperfusion.

Animals with an operation that only exposed the ICA and ECA under the same conditions were used as the sham group (*n* = 8). Animals that underwent ischemia/reperfusion (I/R) were randomly divided into 4 groups (*n* = 8 per group) and intragastric administration (i.g) with desired compounds at a single dose per day: the first group of animals was treated with 1% Tween-80 (vehicle), the second group was treated with 30 mg/kg Nimodipine, the third group was treated with 40 mg/kg PCGE (PCGE 40), and the fourth group was treated with 4 mg/kg PCGE (PCGE 4). All chemicals were administered for 7 days before MCAO and the medication was kept during the experiment until they were sacrificed. Animals underwent 2 h of MCAO followed by reperfusion for 3, 7, or 14 days. The neurologic functions were evaluated by neurobehavioral tests at day 0, 3, 7, and 14 after I/R, and the cognitive functions were measured at days 12 and 13 after I/R. Rats were then anesthetized by chloral hydrate and underwent intracardiac perfusion with paraformaldehyde. The brains were collected for immunohistochemistry.

### 2.3. Neurobehavioral Tests

Two behavioral tests (Longa's scoring and beam walking) were used for assessing the neurofunction of all animals before (baseline) and after I/R (days 3, 7, and 14). According to Longa's scoring [19], the neurological impairment was scored as follows: 0, no neurologic impairment; 1, endoduction of the contralateral anterior limbs and not wholly stretched; 2, circling towards the contralateral side when walked spontaneously; 3, contralateral-side lateriversion when walking; 4, without spontaneous walking and some consciousness lost.

The beam walking which tests animals neurofunctional recovery [20] was assessed on a 6-point scale [21]: 0, balances with steady posture; 1, grasps side of beam; 2, hugs beam and one limb falls down from the beam; 3, hugs beam and 2 limbs fall down from the beam or spins on beam after 60 seconds; 4, attempts to balance on beam but falls off after 40 seconds; 5, attempts to balance on beam but falls off after 20 seconds; and 6, falls off—no attempt to balance or hang onto beam after 20 seconds.

### 2.4. Cognitive Function Test

The cognitive function was evaluated by using a step-down passive avoidance test at day 12 and day 13 after reperfusion. The apparatus comprised a plastic chamber (40 × 20 × 30 cm) with an elevated platform (4.8 × 4.8 × 4.5 cm) placed on the left-side wall. The floor was made of caliber stainless steel bars placed in parallel, 0.5 cm apart. Rats were housed in a dimly lit room for 3 min prior to the experiment. On the first learning day, rats were exposed to a 5 min learning course, during which they were permitted to move freely throughout the chamber prior to being placed on the platform. If the animals stepped down from the platform (i.e., an error trial), they were exposed to an electric foot shock (36 V, AC). The duration from the rats placed on the platform to step down was recorded as latency. After 24 h, the error times and latency were reassessed and recorded as a measure of memory retention.

### 2.5. Histochemistry and Immunohistochemistry

Animals were anesthetized after neurobehavioral tests by intraperitoneal injection of chloral hydrate at days 3, 7, and 14 after I/R and then underwent intracardiac perfusion with 200 ml of 0.01 M phosphate-buffered saline (PBS, pH 7.0), followed by 200 ml of 4% freshly depolymerized paraformaldehyde in 0.1 M phosphate buffer (pH 7.0). The brains were removed and immersed in 4% paraformaldehyde, then were embedded in paraffin and cut into 4 equally spaced coronal blocks. A series of 6 *μ*m thick sections were cut from the blocks and were thaw-mounted onto polylysine-coated slides for hematoxylin-eosin (H&E) staining. For immunostaining, the sections were subsequently boiled in citrate buffer (pH 6.0) within a microwave oven for 5 minutes and washed with PBS (pH 7.0) for 5 minutes followed by quenching with 0.3% H_2_O_2_ in PBS for 20 minutes to block endogenous peroxidase and washed with PBS for three times. Next, the slides were preincubated for 30 minutes in 5% goat serum to block nonspecific binding and then were incubated overnight at 4°C with rabbit polyclonal anti-microtubule-associated protein-2 (MAP2) antibody (dilution 1 : 200; abcam (ab32454), China) to identify neurons and mouse monoclonal anti-glial fibrillary acidic protein (GFAP) antibody (dilution 1 : 200; abcam (ab190288), China) to identify astrocytes, respectively. Slides were washed three times with PBS and then incubated with goat anti-rabbit IgG H&L (dilution 1 : 200; abcam (ab150077), China) or goat anti-mouse IgG (dilution 1 : 200; Millipore (AP124C), China) for 60 minutes. The immunoreactive cells were visualized by the diaminobenzidine reaction after the three-time wash with PBS. Based on a rat brain atlas [22] (George Paxinos and Charles Watson, 2007), immunoreactive cells in the ischemic penumbra and hippocampus were counted with an ocular micrometer (Nikon, Tokyo, Japan) attached to a light microscope at ×400 magnifications. Counting was performed on two consecutive slices, and the mean value for each area was calculated.

### 2.6. Cell Cultures

Primary human astrocytes (Sciencell, HA-1800) were purchased from Beijing Beina Chuanglian Biotechnology Institute, China. Astrocytes were seeded on a collagen-coated 48-well plate or 10 cm dishes. The culture medium (DMEM containing 1 g/l glucose, 4 g/l L-glutamine, 10% FBS, 1.5 g/l sodium bicarbonate, and 1% antibiotic-antimycotic agents) was changed every 48 h after seeding. Cells were grown in a humidified incubator with 95% air-5% CO_2_ at 37°C.

SH-SY5Y cell (ATCC) is a gift from Kunming Institute of Zoology, Chinese Academy of Sciences. Cells were cultured in the same medium as astrocytes. To induce cell differentiation, cells at 80% of confluence were treated with 10 *μ*M retinoic acid dissolved in DMEM with 1% FBS for 3 days as described with modification [23]. Culture medium was replaced every 3 days. The cells were passaged (1 : 12–16) twice a week.

### 2.7. Lactate Dehydrogenase Release

Cell injury was assessed by measuring extracellular lactate dehydrogenase (LDH) release. Primary astrocytes or SH-SY5Y cells were grown to confluence on polycarbonate 48-well plates. In the first set of experiments, astrocytes or SH-SY5Y cells were pretreated with vehicle (DMSO), 15 *μ*g/ml PCGE, 25 *μ*g/ml PCGE, or 50 *μ*g/ml PCGE for 24 h in culture medium before exposure to 0.5 mM H_2_O_2_ in FBS-free DMEM for 4 h. LDH released into the medium was measured using CytoTox 96 Non-Radioactive Cytotoxicity Assay (Promega, Beijing, China) with a microplate reader (DNM-9602G, Beijing Pulang New Technology Company, China) at 493 nm. The amount of LDH released from each test sample was expressed as a percentage of the control and vehicle-treated cells exposed to H_2_O_2_. In the second set of experiments, cells were treated with 25 *μ*g/ml PCGE for 1 to 48 h before exposure to 0.5 mM H_2_O_2_ to examine the time course of protection.

### 2.8. Western Blotting Studies

Western blot analysis was performed as previously described [24]. Cells were harvested after 1 or 24 h treatment with vehicle or PCGE (15, 25 *μ*g/ml). Cells harvested at 1 h were used to examine the nuclear translocation of the nuclear factor erythroid 2-related factor 2 (Nrf2). Nuclear fractions were isolated using a nuclear and cytoplasmic protein extraction kit following the manufacturer's instruction (Beyotime, China). Cells harvested after 24 h were used for whole cell lysate to determine hemeoxygenase 1 (HO-1), NADPH-quinone oxidoreductase (NQO-1), and brain-derived neurotrophic factor (BDNF) levels. 60 *μ*g of total protein of each sample was run through a 10% or 15% SDS-PAGE gel and transferred onto a nitrocellulose membrane (Millipore, China). Membranes were blocked in 5% nonfat dry milk for 1 h and then incubated with polyclonal antibodies (rabbit polyclonal to Nrf2, 1 : 400, Santa Cruz, China; rabbit polyclonal to HO-1, 1 : 500, Proteintech, China; rabbit polyclonal to NQO-1, 1 : 200, Santa Cruz, China; and rabbit polyclonal to BDNF, 1 : 500, Abcam, China) for 2 h. Immunoreactive proteins were visualized by using goat anti-rabbit IgG and enhanced chemiluminescence (EZ-ECL kit, Biological Industries, China) and were then pictured by a gel imaging system (BIO-RAD Gel Doc 2000). The relative densities of Nrf2, HO-1, NQO-1, and BDNF bands were measured and quantified with NIH ImageJ. HO-1, NQO-1, and BDNF were then normalized against *β*-actin. The protein levels were expressed as a percentage of control.

### 2.9. Statistical Analysis

Data from at least eight samples *in vivo* and four independent experiments *in vitro* are presented as means ± SE and were analyzed statistically by one-way analysis of variance (ANOVA) followed by Dunnett post hoc test for comparisons to a single control or Tukey post hoc test for multiple comparisons between groups. A *P* value less than 0.05 is considered statistically significant. All analysis was performed with Prism 5.0 (GraphPad, San Diego, CA).

## 3. Results

### 3.1. PCGE Reversed the Motor Impairments following Cerebral I/R of Rats

**Figure 2:**
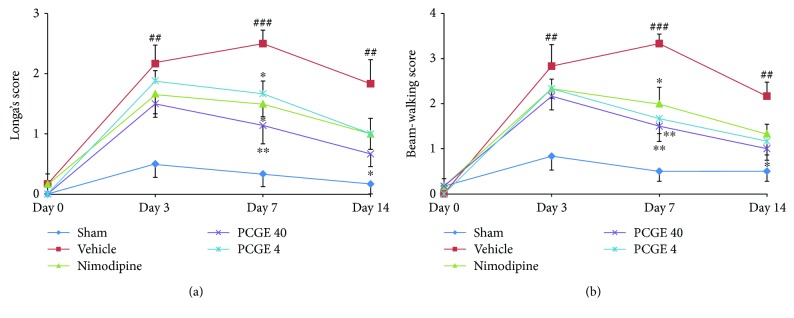
Effects of PCGE on neurological function in cerebral I/R rats. Neurofunctional impairment was scored by Longa's scoring (a), and neurofunctional recovery was scored by the beam-walking test (b). Data are expressed as mean ± SE. *n* = 8. ^##^*P* < 0.01 and ^###^*P* < 0.001 versus the sham group. ^∗^*P* < 0.05 and ^∗∗^*P* < 0.01 versus the vehicle group.

Motor impairment is a common sequela after ischemic stroke. In this study, animal neurofunctional deficit was evaluated with Longa's scoring and neurofunctional recovery was evaluated with beam-walking test before I/R (base line) and at days 3, 7, and 14 after I/R. Compared to the sham group, the vehicle group got a higher neurological score in Longa's test at day 7 (2.50 ± 0.22 versus 0.33 ± 0.21, *P* < 0.001) and day 14 (1.83 ± 0.40 versus 0.17 ± 0.17, *P* < 0.01) after reperfusion (Figure 2(a)), which indicated motor functions were impaired by I/R insult. Compared to the vehicle group, animals that received daily doses of 40 mg/kg PCGE had significantly reduced neurofunctional deficits at day 7 (1.14 ± 0.17 versus 2.50 ± 0.22, *P* < 0.01) and day 14 (0.67 ± 0.21 versus 1.83 ± 0.40, *P* < 0.05) after reperfusion. Similar results were observed in the beam-walking test (Figure 2(b)). Compared to the sham group, the vehicle group had a higher score at day 7 (3.33 ± 0.21 versus 0.50 ± 0.22, *P* < 0.001) and day 14 (2.17 ± 0.31 versus 0.50 ± 0.22, *P* < 0.01), which indicated the impediment of motor functional recovery. The 40 mg/kg PCGE group had a much lower score than the vehicle group at day 7 (1.51 ± 0.34 versus 3.33 ± 0.21, *P* < 0.01) and day 14 (1.00 ± 0.26 versus 2.17 ± 0.31, *P* < 0.05) after reperfusion. The 4 mg/kg PCGE group just improved the neurofunction at day 7 in the beam-walking test.

### 3.2. PCGE Improved the Cognitive Function of Cerebral I/R Rats

**Figure 3:**
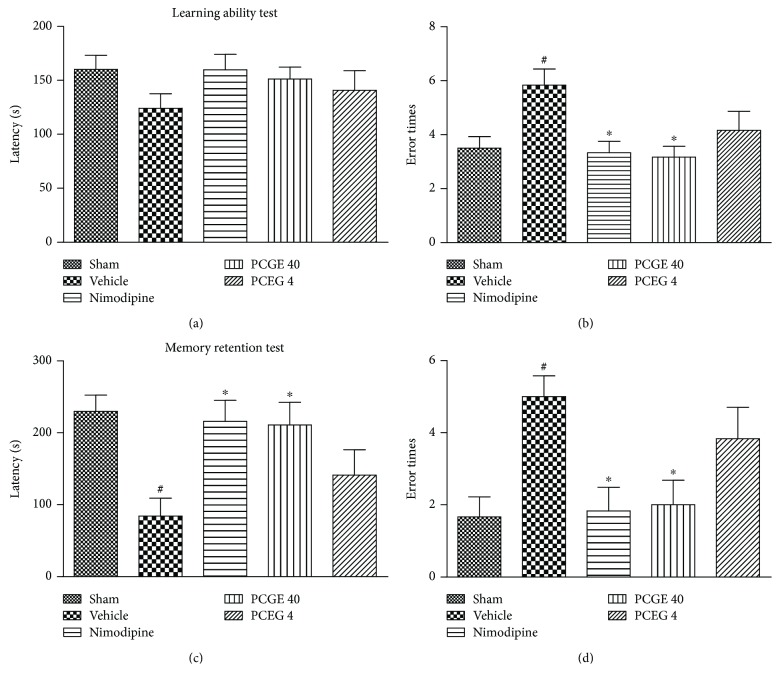
Effects of PCGE on cognitive function in cerebral I/R rats. Learning and memory retention were measured, respectively, at day12 and day 13 after reperfusion by step-down test. In the learning test, longer step-down latency (a) or fewer error times (b) indicate better learning ability. In the memory retention test, longer step-down latency (c) or fewer error times (d) indicate stronger memory retention. Data are expressed as mean ± SE. *n* = 8. ^#^*P* < 0.05 versus the sham group and ^∗^*P* < 0.05 versus the vehicle group.

Cognitive dysfunction is another common sequela of ischemic stroke. The step-down passive avoidance test associates with long-term or reference memory [25] and was used in this study to explore whether a supplement of PCGE would improve the cognitive function in I/R rats. For the learning ability test at day 12 after reperfusion, there was no difference in step-down latency among the groups (Figure 3(a)), but the vehicle group had more error times compared to the sham group (Figure 3(b)). For the memory retention test at day 13 of reperfusion, the vehicle group had a shorter step-down latency (Figure 3(c)) and more error times (Figure 3(d)). These data implied cognitive disorder in the vehicle group. Compared to the vehicle group, the 40 mg/kg PCGE group had less error times both in the learning ability test (3.17 ± 0.40 versus 5.83 ± 0.60, *P* < 0.05) and memory retention test (2.00 ± 0.68 versus 5.00 ± 0.58, *P* < 0.05) and had a longer step-down latency in the memory retention test (210.8 ± 31.4 versus 84.0 ± 25.1, *P* < 0.05), suggesting that a supplement of PCGE could improve learning and memory ability of cerebral I/R rats.

### 3.3. PCGE Alleviated Pathological Lesions in the Penumbra of Cerebral I/R Rats

**Figure 4:**
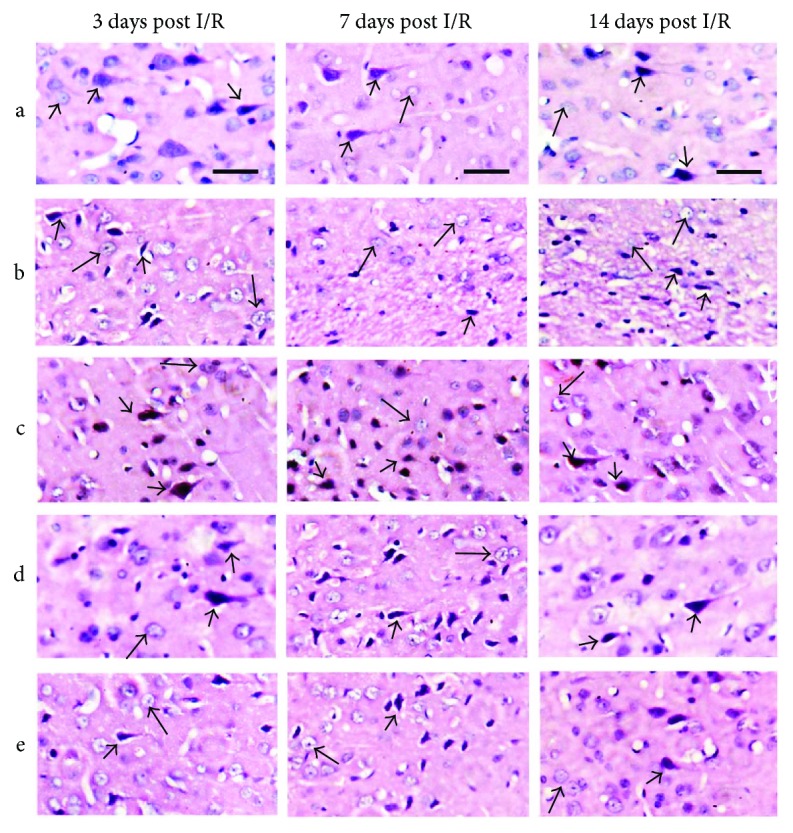
Histological features of the penumbra in cerebral I/R rats (H&E staining): (a) sham group; (b) vehicle group; (c) 30 mg/kg Nimodipine group; (d) 40 mg/kg PCGE group; (e) 4 mg/kg PCGE group. Bar = 20 *μ*m. In the sham group, the cortex showed many normal neurons (arrowheads) and astrocytic nuclei (arrows). In the vehicle group, ischemic penumbra showed shrunken, dense, and angular neurons (arrowheads) and swollen astrocytic nuclei (arrows). Nimodipine or PCGE treatment reduced the pathological lesions in the penumbra of cerebral I/R rats.

The lesions of cortical areas and the CA1 layer of the hippocampus have been known to be the most affected by transient ischemic injury in the brain [26, 27]. Pathological changes in ischemic penumbra were observed by using H&E staining (Figure 4). In the sham group (Figure 4(a)), most neurons in the cortex appeared with normal morphology with well-defined, basophilic nuclei and typical neuritis, and astrocytes were rarely observed. In the vehicle group (Figure 4(b)), at day 3 after reperfusion, neurons in ischemic penumbra were characterized by darkly stained, triangular nuclei and scarcely any cytoplasm as what was reported previously [28] and astrocytes were featured with prominent nuclei and abundant cytoplasm; at day 7 and day 14 after reperfusion, the numbers of neurons in the penumbra dramatically decreased. In the Nimodipine group (Figure 4(c)), 40 mg/kg PCGE group (Figure 4(d)), or 4 mg/kg (Figure 4(e)) PCGE group, neuronal degeneration was obviously alleviated and viable neurons were largely increased compared to the vehicle group.

**Figure 5:**
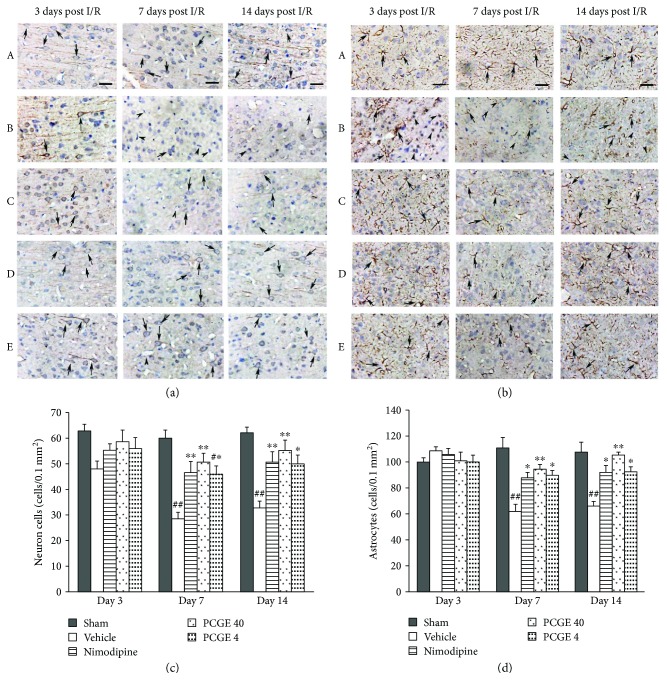
PCGE treatment reduced the damage of neurons and astrocytes in ischemic penumbra. Panel (a) shows normal neurons (arrows) in the sham group or damaged neurons that are shrunken, dense, and angular morphous (arrowheads) in the vehicle group. Panel (b) shows normal astrocytes that are stellate morphous (arrows) in the sham group or damaged astrocytes with pyknosis and are ameboid morphous (arrowheads) in the vehicle group. Nimodipine or PCGE treatment alleviated the morphological damage of neurons and astrocytes at days 7 and 14 after I/R. Bar = 10 *μ*m. Panels (c) and (d) show the effect of PCGE on the numbers of viable neurons (MAP2^+^, 0.1 mm^2^) and astrocytes (GFAP^+^, 0.1 mm^2^) in the penumbra at 3, 7, and 14 days after I/R. Data are expressed as mean ± SE. *n* = 8. ^#^*P* < 0.05 and ^##^*P* < 0.01 versus the sham group; ^∗^*P* < 0.05 and ^∗∗^*P* < 0.01 versus the vehicle group.

The immunohistochemistry result is in accordance with the H&E staining result (Figure 5). MAP2 is required for the morphological differentiation of dendrites of neurons [29, 30]. As shown in Figure 5(a), at day 3 after I/R, there were no notable changes in the intensity and pattern of MAP2-immunopositive (MAP2^+^) cells in all groups. At day 7 and day 14 after I/R, most of the MAP2^+^ dendrites disappeared and the numbers of MAP2^+^ cells were dramatically reduced in the penumbra of the vehicle group (Figure 5(c)), which indicated the most severe injury of the neurons induced by ischemia occurred in this duration. Nimodipine, 40 mg/kg PCGE, or 4 mg/kg PCGE treatment efficiently improved the outcome by increasing the expression of MAP2^+^ dendrites and the number of surviving neurons at day 7 and day 14 after I/R.

GFAP is necessary for the formation of stable glial processes of astrocytes in response to neuronal signals [31]. At day 3 after I/R, increased GFAP immunoreactivity was observed predominantly within the penumbra in all I/R groups (Figure 5(b)). The activation of astrocytes was characterized in morphological changes as hypertrophy, lesser extent proliferation, and increased nuclear size as what was reported previously [32]. Compared to the sham group, at day 7 and day 14 after I/R, most of GFAP-immunopositive (GFAP^+^) processes disappeared and the number of GFAP^+^ cells was markedly decreased in the vehicle group (Figure 5(d)). On the contrary, the pattern and intensity of astrocytes in the Nimodipine or PCGE groups were similar to those in the sham group, the expression of GFAP was slightly upregulated with the intensive foot processes, and the number of surviving astrocytes was increased, which were not statistically different from the sham group.

### 3.4. PCGE Alleviated Pathological Lesions in the CA1 Layer after I/R

**Figure 6:**
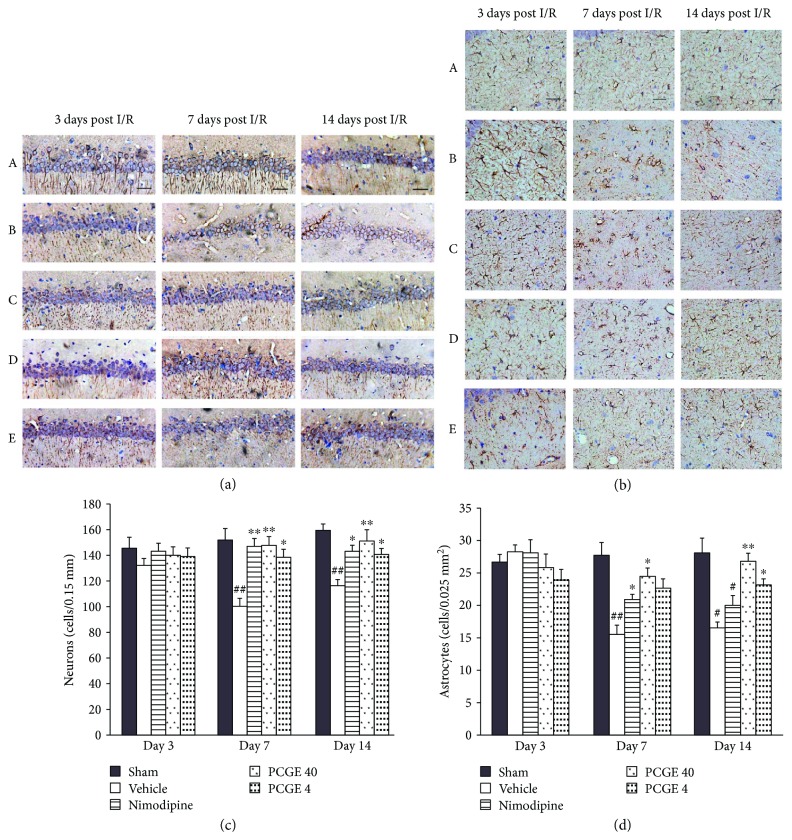
PCGE treatment reduced the damage of neurons and astrocytes in the CA1 layer. Panel (a) shows normal neurons arrayed in order with intact outlines or damaged neurons arrayed asymmetrically in the CA1 layer. Panel (b) shows the normal astrocytes that are stellate morphous or damaged astrocytes that are ameboid morphous with pyknosis in the CA1 layer. Nimodipine or PCGE treatment reduced the damage of neurons and astrocytes at days 3, 7, and 14 after I/R. Bar = 10 *μ*m. Panels (c) and (d) show the effect of PCGE on the numbers of viable neurons (MAP2^+^, 0.15 mm) and astrocytes (GFAP^+^, 0.025 mm^2^) in the CA1 layer at 3, 7, and 14 days after I/R. Data are expressed as mean ± SE. *n* = 8. ^#^*P* < 0.05 and ^##^*P* < 0.01 versus the sham group; ^∗^*P* < 0.05 and ^∗∗^*P* < 0.01 versus the vehicle group.

The damage of neurons and astrocytes in the CA1 area of the hippocampus after I/R is summarized in Figure 6. Compared to the sham group, no obvious density and morphology changes of MAP2^+^ cells were seen in all I/R groups at day 3 after reperfusion. However, at day 7 and day 14 after reperfusion, most of the MAP2^+^ dendrites disappeared, pyramidal neurons were arranged asymmetrically, and the numbers of surviving neurons were dramatically reduced in the vehicle group. Compared to the vehicle group, MAP2^+^ dendrites and the number of surviving neurons were increased and the neuron arrays were lined orderly and intact in the Nimodipine or PCGE groups (Figures 6(a) and 6(c)).

Similarly, as shown in Figures 6(b) and 6(d), compared to the sham group, GFAP immunoreactivity increased in all I/R groups at day 3 after reperfusion. At day 7 and day 14 after reperfusion, while the expression of GFAP^+^ processes and the numbers of surviving astrocytes were reduced in the vehicle group, the GFAP^+^ processes and the number of astrocytes were increased in the Nimodipine or PCGE groups compared to the vehicle group.

### 3.5. PCGE against H_2_O_2_-Induced Brain Cell Damage

**Figure 7:**
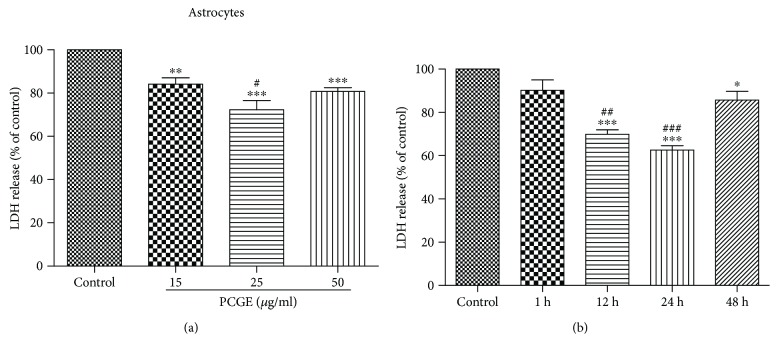
Effects of PCGE on H_2_O_2_-induced LDH release from human primary astrocytes. (a) Cells were preincubated for 24 h with the different concentrations of PCGE (15, 25, and 50 *μ*g/ml) and then exposed to 0.5 mM of H_2_O_2_ for 4 h in FBS-free medium. ^∗∗^^,^^∗∗∗^Significant differences at the *P* < 0.01 and *P* < 0.001 levels, respectively, versus the control group; ^#^*P* < 0.05 versus the 15 *μ*g/ml group. (b) LDH release from astrocyte cells that were pretreated with 25 *μ*g/ml PCGE for different durations (up to 48 h) before exposure to 0.5 mM H_2_O_2_. ^∗^^,^^∗∗∗^Significant differences at the *P* < 0.05 and *P* < 0.001 levels, respectively, versus the control group; ^##^*P* < 0.01 and ^###^*P* < 0.001 versus the 1 h group. The results were quantified by using an LDH activity kit assay and expressed as percentages of LDH released in PCGE-treated or vehicle-treated cells (control) exposed to H_2_O_2_. Values are means ± SE; *n* = 5 independent experiments. ANOVA followed by the Tukey post hoc test.

LDH release is an indicator of cell membrane damage and death. Compared to control, 24 h pretreatment with 15, 25, and 50 *μ*g/ml PCGE significantly reduced H_2_O_2_-induced cell death in astrocytes to 84 ± 6% (*P* < 0.01), 72 ± 10% (*P* < 0.001), and 81 ± 4% (*P* < 0.001), respectively, as shown in Figure 7(a). A time course study showed that pretreatment with 25 *μ*g/ml PCGE for 12, 24, and 48 h reduced astrocyte death to 70 ± 5% (*P* < 0.001), 63 ± 5% (*P* < 0.001), and 86 ± 9% (*P* < 0.05), respectively, compared to the control (Figure 7(b)).

**Figure 8:**
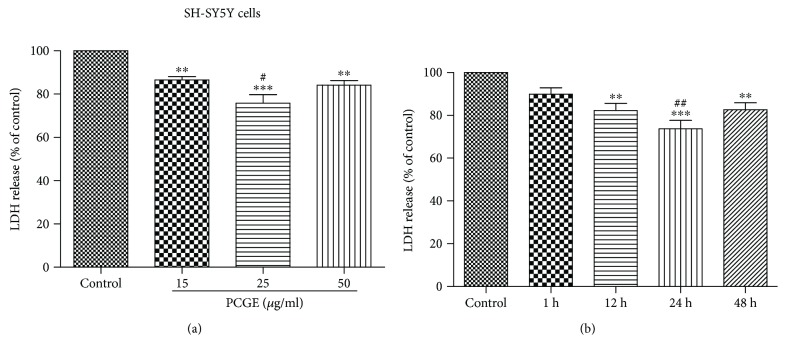
Effects of PCGE on H_2_O_2_-induced LDH release from SH-SY5Y cells. (a) Cells were preincubated for 24 h with the different concentrations of PCGE (15, 25, and 50 *μ*g/ml) and then exposed to 0.5 mM of H_2_O_2_ for 4 h in FBS-free medium. ^∗∗^^,^^∗∗∗^Significant differences at the *P* < 0.01 and *P* < 0.001 levels, respectively, versus the control group; ^#^*P* < 0.05 versus the 15 *μ*g/ml group. (b) LDH release from SH-SY5Y cells that were pretreated with 25 *μ*g/ml PCGE for different durations (up to 48 h) before exposure to 0.5 mM H_2_O_2_. ^∗∗^^,^^∗∗∗^Significant differences at the *P* < 0.01 and *P* < 0.001 levels, respectively, versus the control group; ^##^*P* < 0.01 versus the 48 h group. The results were quantified by using an LDH activity kit assay and expressed as percentages of LDH released in PCGE-treated or vehicle-treated cells (control) exposed to H_2_O_2_. Values are means ± SE; *n* = 5 independent experiments. ANOVA followed by the Tukey post hoc test.

Similar effects of PCGE on H_2_O_2_-induced damage in SH-SY5Y cells were observed. Compared to the control, 24 h pretreatment with 15, 25, and 50 *μ*g/ml PCGE significantly reduced cell death to 87 ± 3% (*P* < 0.01), 76 ± 9% (*P* < 0.001), and 84 ± 5% (*P* < 0.01), respectively, (Figure 8(a)). A time course study showed that pretreatment with 25 *μ*g/ml PCGE for 12, 24, and 48 h reduced cell death to 82 ± 7% (*P* < 0.01), 74 ± 9% (*P* < 0.001), and 83 ± 7% (*P* < 0.01), respectively, compared to the control (Figure 8(b)).

LDH results indicated that 24 h pretreatment with 25 *μ*g/ml PCGE had the best effect on protecting brain cells against oxidative stress-induced damage.

### 3.6. Activating Nrf2 in Brain Cells by PCGE

**Figure 9:**
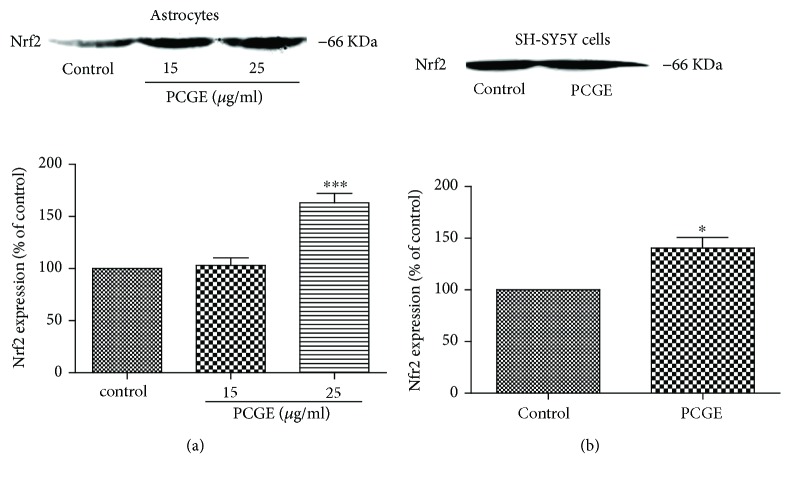
Western blots for Nrf2 accumulation in nuclei of astrocytes and SH-SY5Y cells. (a) Protein was prepared from primary astrocytes in the absence (control) or presence of PCGE (15 and 25 *μ*g/ml) for 1 h treatment. (b) Protein was prepared from SH-SY5Y cells in the absence (control) or presence of 25 *μ*g/ml PCGE for 1 h treatment. Histograms show the mean optical density (OD) for a total of 4 samples for each group. Error bars show SE. *P* < 0.05 or *P* < 0.001 is indicated by ∗ or ∗∗∗ versus the control group.

Nrf2 plays a pivotal role in the cellular defense system against oxidative stress. Under oxidative stress status, Nrf2 is released from Keap1 in cytoplasm and transferred into the nucleus to induce antioxidative response. Possible activation of Nrf2 by PCGE was investigated by Western blot. Nuclear Nrf2 accumulation after 1 h treatment with 25 *μ*g/ml PCGE was 1.63 ± 0.16-folds higher (*P* < 0.001) in astrocytes (Figure 9(a)) and 1.40 ± 0.16-folds higher (*P* < 0.05) in SH-SY5Y cells (Figure 9(b)) compared to that in the control.

### 3.7. Upregulating Nrf2 Downstream Protein in Brain Cells by PCGE

**Figure 10:**
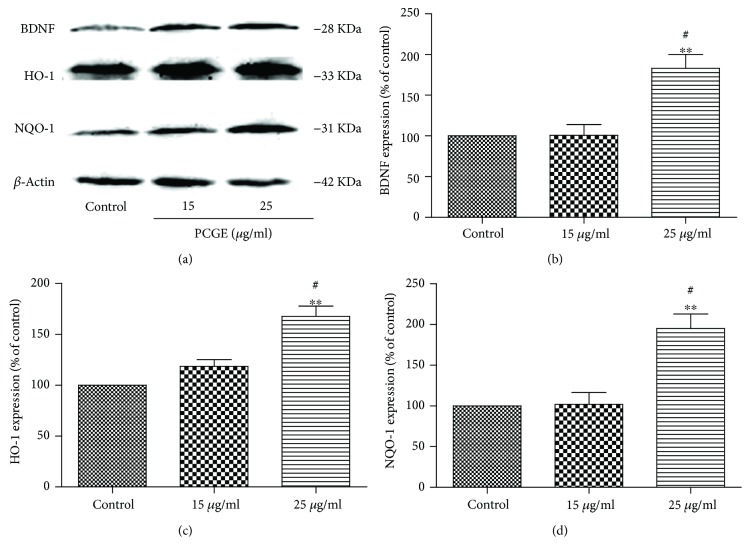
Western blots for BDNF, HO-1, and NQO-1 expressions in astrocytes. Protein was prepared from primary astrocytes in the absence (control) or presence of PCGE (15 and 25 *μ*g/ml) for 24 h treatment. Histograms show the mean optical density (OD) for each band expressed relative to the corresponding *β*-actin levels for each sample, for a total of 4 samples for each group. Error bars show SE. *P* < 0.01 is indicated by ∗∗ versus the control group, ^#^*P* < 0.01 versus the 15 *μ*g/ml group.

**Figure 11:**
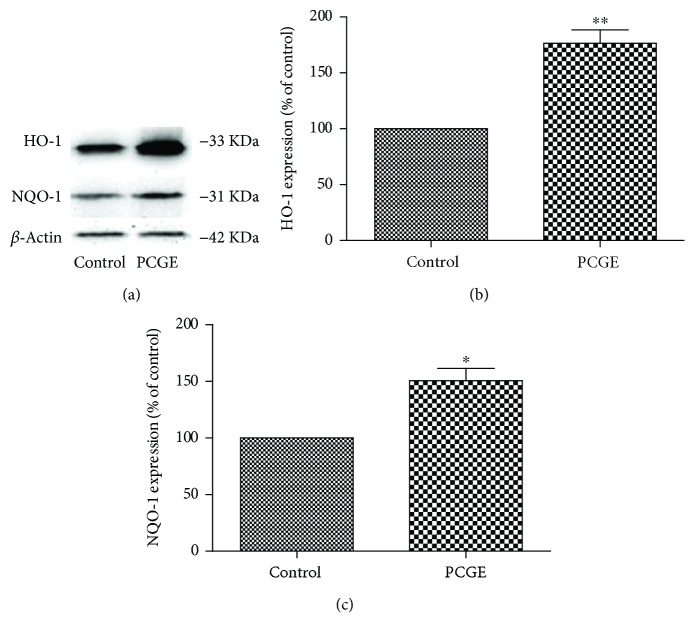
Western blots for HO-1 and NQO-1 expressions in SH-SY5Y cells. Protein was prepared from SH-SY5Y cells in the absence (control) or presence of PCGE (25 *μ*g/ml) for 24 h treatment. Histograms show the mean optical density (OD) for each band expressed relative to the corresponding *β*-actin levels for each sample, for a total of 4 samples for each group. Error bars show SE; *P* < 0.05 or *P* < 0.01 is indicated by ∗ or ∗∗ versus the control group.

After being transferred into the nucleus and combining with the antioxidative response element (ARE), Nrf2 starts the transcription of phase II antioxidant enzymes, such as BDNF (a growth factor that promotes neuronal survival), HO-1 (an enzyme that catalyzes the degradation of heme), and NQO-1 (a ubiquitous flavoenzyme that catalyzes the two-electron reduction of quinones to hydroquinones). Compared to the control, BDNF, HO-1, and NQO-1 in astrocytes were 1.83 ± 0.29-folds (*P* < 0.01), 1.67 ± 0.17-folds (*P* < 0.01), and 1.95 ± 0.31-folds (*P* < 0.01) increased, respectively, after 24 h treatment with 25 *μ*g/ml PCGE (Figure 10). HO-1 and NQO-1 in SH-SY5Y were also 1.56 ± 0.12-folds (*P* < 0.01) and 1.51 ± 0.11-folds (*P* < 0.05) increased, respectively, after 24 h treatment with 25 *μ*g/ml PCGE, compared to the control (Figure 11).

## 4. Discussion

**Figure 12:**
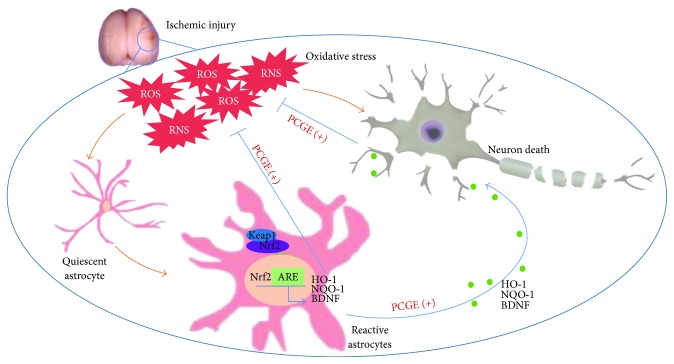
The protective effects and related mechanisms of PCGE on ischemic stroke. After cerebral ischemia, the allegro accumulation of ROS and RNS results in damage of neurons and astrocytes. Under status of oxidative stress, Nrf2 is released from Keap1 in cytoplasm and transferred into the nucleus, whe re it combines to ARE and upregulates antioxidant genes such as HO-1, NQO-1, and BDNF against the oxidation-mediated neurotoxicity. PCGE can stimulate endogenous antioxidative responses in astrocytes and neurons, facilitating reactive astrocytes providing antioxidants and neurotrophic factor to neurons, thereby attenuating neuronal damage.

Production of excessive oxidant ROS/RNS is a fundamental mechanism of cell damage following cerebral ischemia. The present study aimed to explore the oxidative stress-related mechanisms of PCGE on ischemic stroke. Our data demonstrated, for the first time, that PCGE exerts neuroprotective effect in ischemic stroke by activating cellular antioxidant systems (Figure 12).

Ischemic stroke-induced cerebral damage includes the core of necrotic cell death and the ischemic penumbra. Although it is impossible to rescue the neurons in the infarction core, the penumbra, which surrounds the damaged core, is considered as a target for acute intervention in ischemic stroke. The development of the penumbra is a time-related process where cells will die in the following hours to days. These lesions of the penumbra constitute approximately 10% of all brain infarcts [33]. Therefore, early management of these areas will be giving rise to an overall good prognosis [34]. Calcium channel blockers can reduce free radical stress to arrest the neural cell death and alleviate the damage after ischemic stroke [35–37]. Thus, Nimodipine was used as a positive control in this study. Corresponding with what was reported previously [38], data from this present study showed that from 7 to 14 days after cerebral I/R, the number of neurons in the penumbra were largely reduced with damaged morphology in the vehicle group. Simultaneously, severe motor impairments were observed in this duration, implying that the damage and death of neurons contribute to the neurofunctional deficits. The damage of neurons in the penumbra is functionally inactive but is still viable and reversible [39–41]. During this acute phase, quiescent astrocytes are activated into reactive astrocytes, which act as a guardian that defends neurons from insult via transporting/metabolizing various molecules for supplying nutrition and improving cell-to-cell signaling and neurotransmission, which contributes to neurological recovery after stroke [42, 43]. A failure to accomplish these functions may constitute a major pathogenic component in stroke [44] and result in selective neuronal necrosis. Interestingly, in this study, astrocytes in the penumbra were activated in all animals at day 3 after reperfusion. Thereafter, most of the astrocytes died from 7 days to 14 days after reperfusion in the vehicle group, which could be a reasonable account for the neuron death and neurofunctional deficits in these animals. Oppositely, PCGE alleviated neuronal damage and increased viable neurons in the penumbra in this duration. A possible explanation for these results may be the lightly elevated activation of astrocytes and the retarded death of astrocytes by PCGE. At 7 days to 14 days after reperfusion, GFAP^+^ processes were increased in PCGE groups, which might improve astrocyte response to neuronal signals. Meanwhile, the multitudinous surviving astrocytes in the PCGE groups might be conducive to neuronal survival. Consistent with these morphological results, neurological results of the long-term test showed that PCGE mitigated neurological deficit and facilitated neurofunctional recovery at day 7 and day 14 after reperfusion. Previous work of our lab has proved that PCGE can reduce the infarction area of cerebral I/R rats [45]. The pathological and behavioral data from this present study indicated that PCGE could alleviate pathological lesions in the penumbra by avoiding or reducing neuronal degeneration and thereupon improve the prognosis of cerebral I/R rats. At 2 weeks after I/R, it was noted that animals in the vehicle group also showed an improved behavior mildly, and the reason for this could partially be the formation of collateral circulation and functional compensation after brain injury [46].

Besides infarction core, the CA1 region of the hippocampus also is particularly vulnerable to ischemia insult, and delayed neuron death occurred there within minutes after transient cerebral ischemia that can lead to cognitive dysfunction [47–49]. In this study, the number of neurons and astrocytes in the CA1 layer was markedly reduced and the animals exhibited more error times in learning and memory retention tests from 7 to 14 days after I/R in the vehicle group, implying the learning and memory dysfunction. PCGE treatment increased viable neurons and astrocytes in the CA1 layer and kept the neurons in alignment. In the cognitive trial, the PCGE group made fewer error times and had longer latency than those of the vehicle group. These findings illustrated that PCGE have distinct functions in redressing the delayed neuron death in the hippocampus, thereby repairing the cognitive dysfunction.

It is well known the role of oxidative stress in neuron death after cerebral ischemia. In the normal condition, ROS are a physiological product and are neutralized by endogenous antioxidant defense mechanisms. After cerebral ischemia, oxidants are overproduced because the redox in the natural endogenous antioxidant system is in disequilibrium and the detoxification mechanisms are inactivated. One of the most important pathways of the cellular defense system responding to oxidative stress is to urge Nrf2 transfer into the nuclei, where it binds to the ARE, activates a myriad of genes including HO-1 and NQO-1, and provides efficient cytoprotection against oxidative stress [50–52]. In this study, pretreatment with 25 *μ*g/ml PCGE for 24 h gave rise to the most remarkable protection to SH-SY5Y cells from H_2_O_2_-induced damage. This protective effect may be attributed to the increased Nrf2 accumulation in nuclei and upregulated HO-1 and NQO-1 expressions. These results indicated that PCGE might stimulate the transcription of Nrf2 to steadily uplift the basic Nrf2 antioxidant system and thereby protect neurons against toxicity created by oxidative stress.

Astrocytes, rich in certain antioxidants, appear essential to maintain cerebral antioxidant competence and shelter neurons from superoxide-induced deleterious effects [6, 53, 54]. Activation of the Nrf2/ARE pathway, especially in astrocytes, has been established to protect the brain against neurotoxicity from a variety of insults [55]. The present study showed that PCGE reduced astrocyte damage induced by H_2_O_2_, increased Nrf2 accumulation in nuclei, and upregulated HO-1 and NQO-1 expressions in astrocytes. Besides antioxidation, astrocytes release nutrients and neurotrophins to support the neurons against oxidative stress [56]. BDNF, an Nrf2-dependent growth factor [57], was also upregulated in astrocytes after being treated with PCGE. BDNF promotes the neuronal survival, differentiation, and synaptic plasticity. It has been reported that BDNF exerts vigorous effect on ischemic tissue [58] and presents a promising approach to enhance the neurofunctional recovery after brain injury [59–62]. The early recovery of neurofunction in ischemic brain has been considered as the neuroprotection of BDNF upregulation instead of differentiation from the stem cell [63]. PCGE may, therefore, promote neuronal survival by providing the phase II antioxidant enzymes and neurotrophic factor to neurons by activating the astrocyte-mediated antioxidative pathway. All the results above logically explained the increased surviving neurons and the facilitated neurofunction recovery in the PCGE groups.

Compounds activating the Nrf2 pathway and modulating HO-1, NQO-1, and BNDF expressions have been reported to protect the neurons from a variety of injury [64–66] and have been proposed to serve as an important candidate for therapeutic strategy aimed at limiting neural degeneration and improving neurological recovery. The present study showed that PCGE could protect the brain cells from oxidative stress by activation of the Nrf2/ARE pathway and these *in vitro* results of PCGE were in accordance with its benefit on the restoration of neurofunction *in vivo*. All the results from this study indicated that early treatment with PCGE might provide long-term benefits for neurofunctional recovery after cerebral I/R. It would be interesting to explore the combination of PCGE with existing neuroprotectants or thrombolytics in future research.

## 5. Conclusions

In conclusion, this study demonstrated that PCGE was effective in reducing neurotoxicity in ischemic stroke associated with decreasing H_2_O_2_-induced LDH release, upregulating HO-1, NQO-1, and BDNF expressions through activation of Nrf2 in brain cells. These results support the hypothesis that PCGE stimulates endogenous antioxidative responses in brain cells, thereby protecting the neurons and improving prognosis after ischemic stroke. Therefore, PCGE could be a potential therapeutic candidate for ischemic stroke and other oxidative stress-associated neurological disorders.

## Figures and Tables

**Table 1:** Contents of phenolics in PCGE. The contents of different compounds in PCGE as determined by HPLC (see Figure 1).

Peak	Rt (min.)	Compound	Content (%)
1	6.47	Gastrodin	0.36
2	13.78	4-Hydroxybenzylalcohol	3.27
3	29.48	3,4-Dihydroxybenzaldehyde	0.208
4	48.60	4-Hydroxybenzaldehyde	2.85

Rt = retention time.
